# Anatomical Insights Into the Equine Cervical Intervertebral Foramina: Implications for Veterinary Practice

**DOI:** 10.1111/ahe.70154

**Published:** 2026-07-15

**Authors:** Renato Lopes Previdelli, Rupert Frederick Dash, Catrin Sian Rutland, Rachel Tucker, Alex Hawkins

**Affiliations:** ^1^ Royal Veterinary College University of London London UK; ^2^ School of Veterinary Medicine and Science University of Nottingham Sutton Bonington UK; ^3^ Liphook Equine Hospital Hampshire UK

**Keywords:** applied anatomy, cervical vertebral anatomy, equine surgery, spinal nerves, veterinary surgery

## Abstract

The equine cervical intervertebral foramina (IVF) represent vital anatomical regions facilitating the passage of spinal nerves and associated vasculature. An in‐depth understanding of their anatomy is imperative for the safe implementation of diagnostic and surgical interventions. Despite their clinical significance, anatomical descriptions of the equine cervical IVF remain under‐characterised in the existing literature, particularly in relation to the needs of equine veterinary surgeons. Our study provides comprehensive anatomical analysis of the IVF at the cervical vertebral levels C5/C6 and C6/C7 in the domestic horse ( *Equus caballus* ), targeting advancing clinical methodologies pertinent to cervical foraminotomy procedures. A series of anatomical dissections on equine cadavers' necks was performed to comprehensively evaluate the external musculature and establish contextual orientation, subsequently leading to more intricate dissections and descriptions. This study aimed to undertake descriptive anatomical dissections of the C5/C6 and C6/C7 IVF, employing direct observation of clinical and surgical importance. Data analysis included visual documentation with development of annotated diagrams to elucidate the spatial relationships between skeletal, muscular, neural, and vascular structures within and surrounding the C5/C6 and C6/C7 foramina. The results present a final, sequential, detailed anatomical characterisation of the IVF at the specified cervical levels, highlighting morphological descriptions, structural orientation, and adjacent tissue planes. Critical features relevant to clinical practice are delineated, notably the proximity of neurovascular structures to bony landmarks frequently encountered during surgical interventions. The descriptive anatomy in this study provides novel anatomical interpretations of the equine cervical IVF region and advocates for safer, evidence‐based clinical practices in managing conditions that require clinical and surgical interventions.

## 1 Introduction

Understanding the anatomy of the intervertebral foramina (IVF) in horses ( *Equus ferus caballus* ) is crucial for the equine veterinarian, especially considering the clinical manifestations that can arise from nerve compression in this region. The IVF serve as a pivotal passageway for cervical spinal nerves, which may become compromised due to conditions such as intervertebral disc disease or bony impingement, as a result of articular process joint enlargement or osteoarthritis. Such compression can lead to significant neurological deficits, adversely affecting the quality of life for affected horses. A notable percentage of horses presenting with lameness exhibit signs of cervical compressive myelopathy or pain associated with IVF compromise (Sioutas and Kapetanakis 2016). These conditions not only impair locomotion but can also lead to chronic pain syndromes, necessitating advanced surgical intervention. Case reports in veterinary medicine show that complications arising from poorly executed surgical techniques in the intervertebral cervical area, such as inadvertent nerve damage or incomplete decompression, further complicate the management of these cases (Elliott and Boucher 2018). Thus, a thorough understanding of the anatomical relationships and potential complications associated with cervical IVF is essential for equine surgeons. By enhancing their comprehension of this region, equine practitioners can improve their surgical approaches, better manage the conditions affecting the IVF, and consequently enhance the recovery and quality of life for horses suffering from nerve compression syndromes (Long et al. 2022).

**FIGURE 1:**
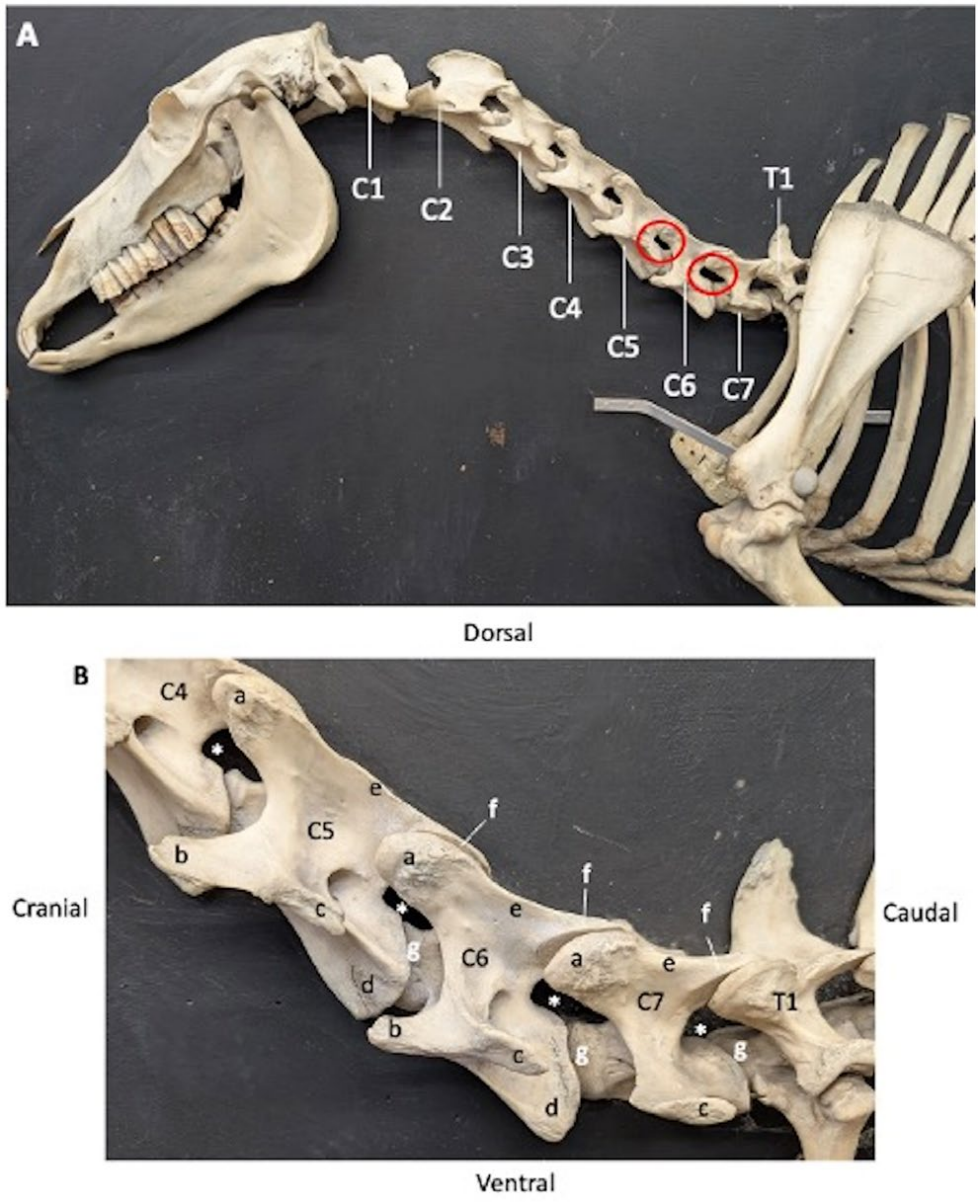
Equine cervical vertebrae, left lateral view. (A) C1/Atlas‐C7. Red circles represent the intervertebral foramen areas of C5–C6 and C6–C7, respectively (Singh 2018). (B) C5–C7. C4–C7 & T1 labels indicate the body of the vertebrae; a: cranial articular process; b: ventral process; c: transverse process; d: ventral process; e: spinous process; f: caudal articular process; g: vertebral joint surface; *: intervertebral foramen space (Pasquini 1991).

In equines, the cervical vertebrae exhibit distinct morphological characteristics to meet their quadrupedal locomotion and overall anatomical function. Comprising seven cervical vertebrae (C1, or Atlas, through to C7; as depicted in Figure 1A), the cervical column in equids is notably different from that of bipedal species in terms of alignment and mobility. The equine cervical spine is horizontally aligned, supporting the weight of the head in a manner that facilitates extensive mobility at the cranial cervical region, particularly at the atlanto‐occipital (C1, or Atlas) and atlantoaxial (C2, or Axis) joints. This contrasts with the more constrained mobility observed in the caudal cervical spine (C3 to C7) (Bosch et al. 2025).

Anatomically, the cervical vertebra consists of a vertebral body, a vertebral arch, and various processes (Figure 1B). The transverse processes are significant in horses, as they extend laterally and house the transverse foramina, which accommodate the vertebral arteries (Gilchrist 2002; Dias et al. 2024). The spinous processes, particularly on C6 and C7, are notably longer than those of the other cervical vertebrae and provide attachment points for muscles and ligaments (Gilchrist 2002; Dias et al. 2024). Each vertebra connects through synovial joints, known as the articular process joints, which are susceptible to various morphological variations and degenerative changes, such as osteoarthritis, affecting their functionality (Rombach et al. 2014).

**FIGURE 2:**
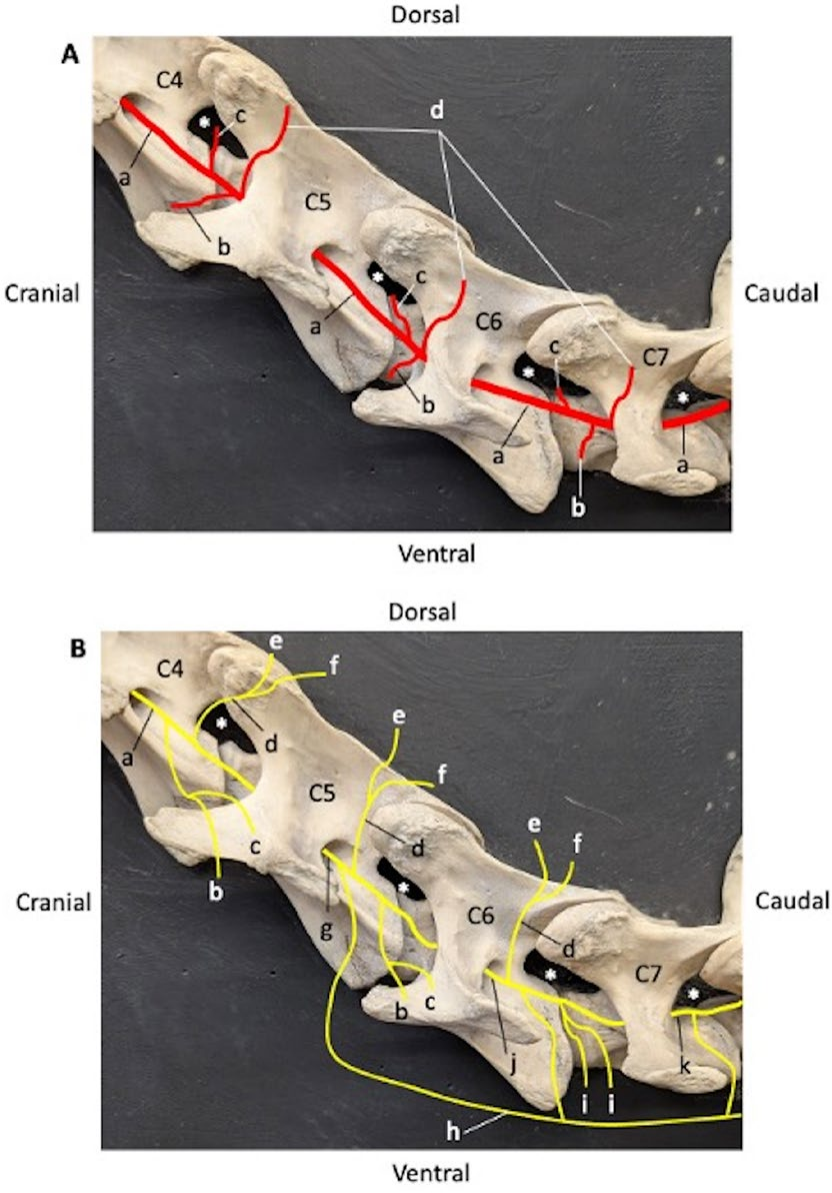
Anatomical representation of important arteries and nerves in the region of equine cervical vertebrae C5–C7, left lateral view. C4–C7 & T1 labels indicate the body of the vertebrae. (A) Arteries of importance. a: vertebral artery; b: ventral muscular branch; c: spinal branch; d: dorsal muscular branch; *: intervertebral foramen space (Schmaltz 1928; Pasquini 1991). (B) Nerves of importance a: spinal nerve C4; b: ventral branch; c: cutaneous branch; d: dorsal branch; e: medial branch of the dorsal branch; f: lateral branch of the dorsal branch; g: spinal nerve C5; h: phrenic nerve; i: supraclavicular (cutaneous) branches; j: spinal nerve C6; k: spinal nerve C7*: intervertebral foramen space (Pasquini 1991).

Formed by the cervical vertebrae, the equine cervical region is a complex anatomical region where the IVF play pivotal roles in the transmission of spinal nerves and vascular structures, making it a critical area for both diagnosis and surgical interventions (Figures 1 and 2). The foramina between the cervical vertebrae C5–C6 and C6–C7 are particularly important due to their association with common equine conditions such as cervical stenotic myelopathy and radiculopathy, which necessitate surgical intervention to alleviate nerve compression and restore function (Corraretti et al. 2020). Despite the existing referencing of surgical approaches, a comprehensive anatomical description of these areas specifically tailored to the surgical anatomy of the equine IVF in this context is notably lacking.

The vasculature of the equine IVF is a compact, multi‐source network comprising radicular arteries and veins that accompany the exiting spinal nerve roots and form connections with the surrounding vertebral and epidural venous systems (Figure 2A). The distal radicular arteries derive from segmental and epidural arteries, delivering blood to dorsal and ventral roots as they exit the IVF, where venous drainage occurs through the dorsal and ventral radicular veins, similarly to the anatomical organisation of arteries, into the vertebral venous plexuses and spinal venous network (Pasquini 1991; Prange et al. 2015; Wood et al. 2021). In equids, pathologic narrowing or osteophytic remodelling of the articular processes of the cervical vertebrae can compress the IVF contents and cause local damage, which further highlights the importance of vascular considerations in cervical thoracolumbar disease and radiculopathy in horses (Haussler et al. 2019; Prange et al. 2015).

The exiting nerve roots are closely associated with adjacent vertebral vessels (Figure 2B); thus, ultrasound‐guided injections in this region must consider the innervation of the IVF and avoid the vertebral artery and venous structures to prevent intravascular injection or hematoma (Wood et al. 2021; Fouquet et al. 2022).

**FIGURE 3:**
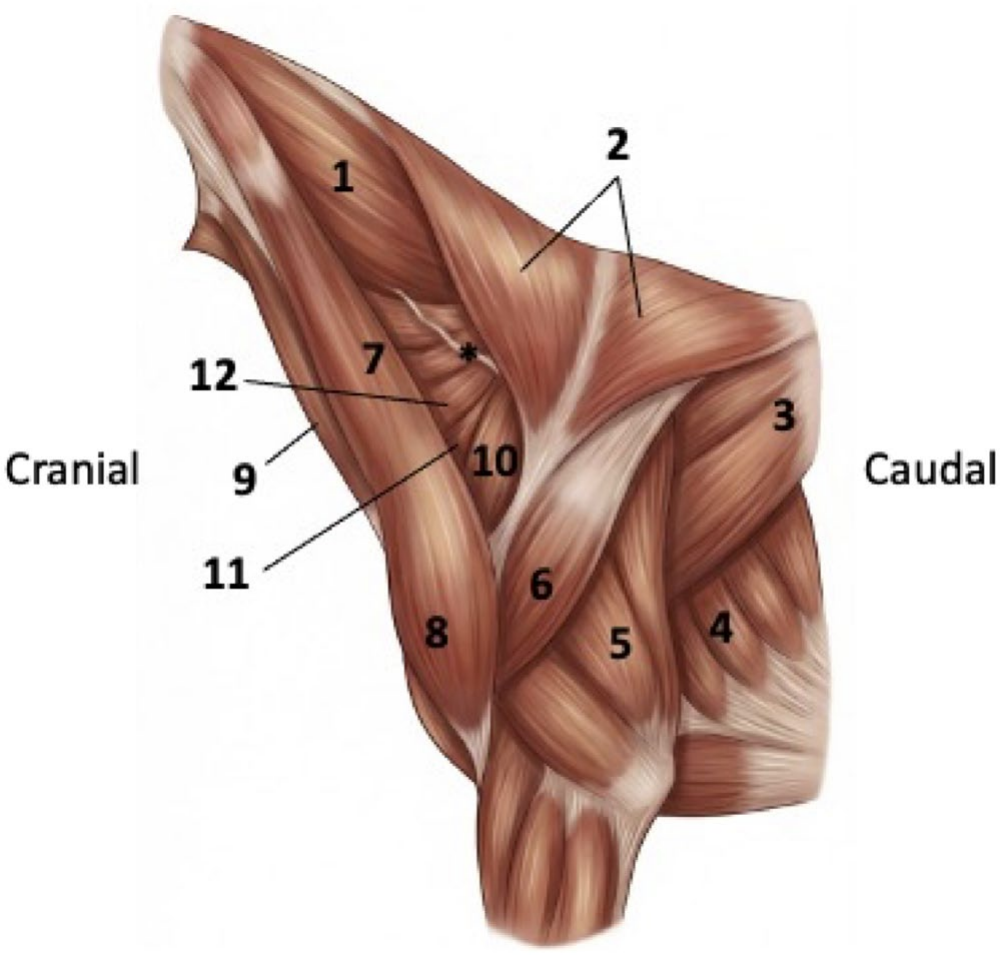
Schematic representation of the musculature of the equine neck and cranial thorax, left lateral view of the superficial musculature after skin removal. 1: Splenius muscle; 2: Trapezius muscle (cervical and thoracic parts); 3: Latissimus dorsi muscle; 4: Serratus ventralis thoracis muscle; 5: Long head of the triceps muscle; 6: Deltoideus muscle; 7: Omotransversarius muscle; 8: Brachiocephalicus muscle; 9: Sternocephalicus (sternomandibularis) muscle; 10: Supraspinatus muscle; 11: Subclavius muscle; 12: Serratus ventralis cervicis muscle; *: spinal accessory muscle (Adapted from Pasquini 1991, schematic illustration created using Ilustrae).

Before accessing the IVF at the level of C5–C7, veterinary surgeons must consider the muscular anatomy that overlies the skeletal structures and the deeper vessels and nerves. The superficial and deep neck musculature of the horse form a layered system that stabilises and mobilises the cervical spine during movement. Superficial muscles, including the trapezius, splenius capitis, and sternocephalicus, span the neck and contribute to head carriage, forelimb propulsion, and gross movements of the head and neck, while providing substantial force to elevate or retract the neck in locomotion (Pepe et al. 2014) (Figure 3).

**FIGURE 4:**
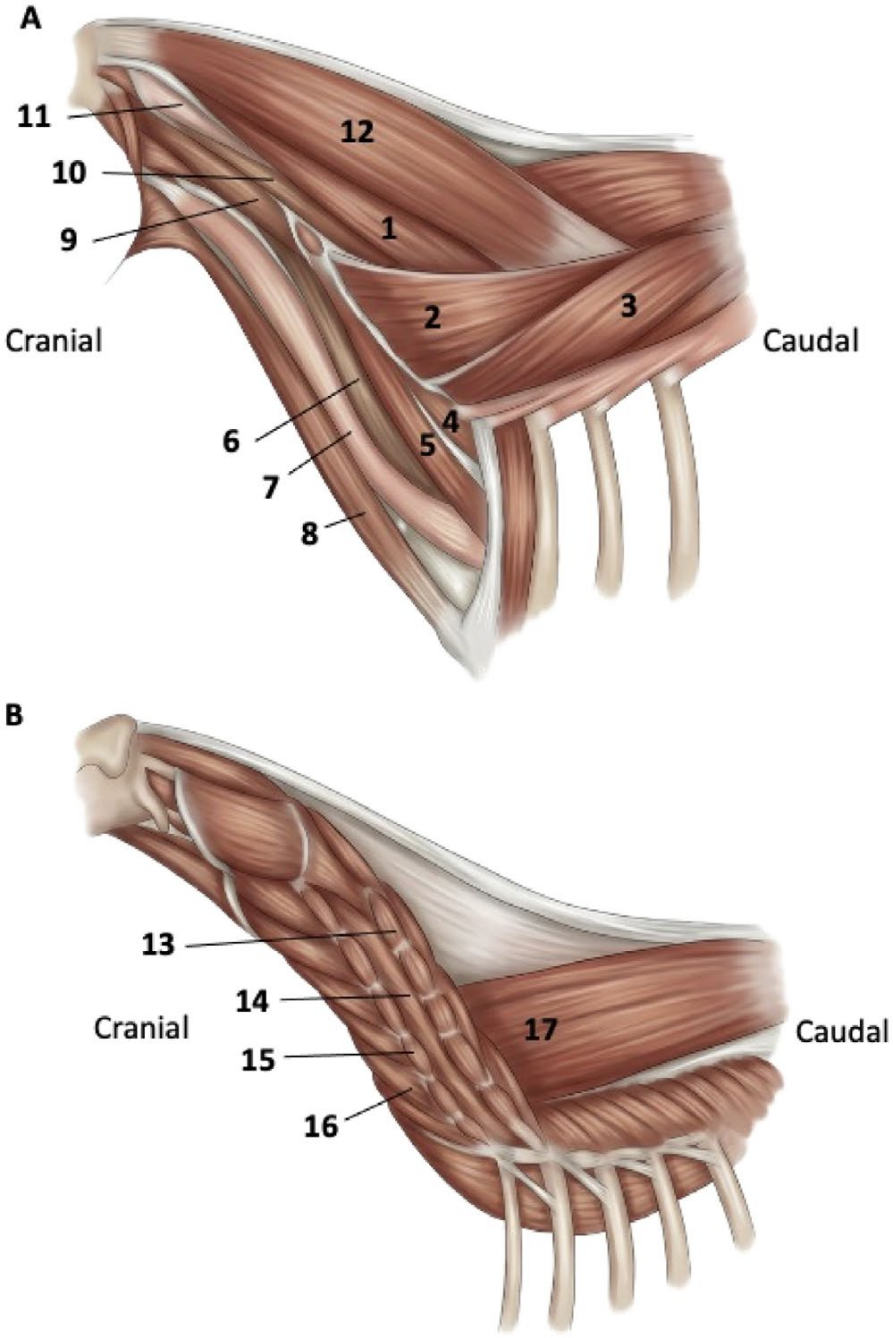
Schematic representation of the deep musculature of the equine neck, left lateral view. (A) Deep musculature after removal of left front limb, splenius muscle, sternocephalicus muscle, omohyoideus muscle, and serratus ventralis cervicis muscle. (B) Deep dissection with deepest layers of muscle before the vertebrae. 1: Longissumus capitis muscle; 2: Longissimus cervicis muscle; 3: Longissimus thoracis muscle; 4: Ventral part of the scalenus medius muscle; 5: Dorsal part of the scalenus medius muscle; 6: Oesophagus; 7: Trachea; 8: Sternohyoideus muscle; 9: Longus capitis muscle; 10: Longissimus atlantis muscle; 11: Attachment of longissimus atlantis muscle onto atlas; 12: Semispinalis capitis muscle; 13: Multifidus muscle; 14: Intertransversarii dorsalis cervicis muscle; 15: Intertransversarii ventralis cervicis muscle; 16: Longus colli muscle; 17: Spinalis cervicalis muscle (Adapted from Pasquini 1991, created using Ilustrae).

Deep segmental muscles, such as the longus colli and the multifidus cervicis muscles, lie closest to the vertebral column and offer proprioceptive feedback and fine stabilisation at each intervertebral level, counteracting flexion, extension, and rotational stresses to protect the cervical joints (Clayton et al. 2010; Pepe et al. 2014). The balance between these layers provides dynamic stability, with deep muscles acting as stabilisers and superficial muscles supplying larger excursions (Clayton et al. 2010; McElroy et al. 2019) (Figure 4).

Previous studies have explored various aspects of the cervical spine anatomy in equines; however, most have focused on broader anatomical characteristics of such important structures considering their details for equine surgical applications. Furthermore, while approaches such as the extraforaminal ligament attachments have been evaluated in human spinal anatomy (Kraan et al. 2009), their counterparts in veterinary medicine, especially in horses, remain largely unexplored. This creates a relevant need in the literature, particularly regarding the practical surgical guidelines that equine surgeons require when dealing with surgical approaches and their challenges related to the C5–C6 and C6–C7 IVF.

The primary aim of this descriptive research study was to present an innovative and descriptive anatomical representation of the equine IVF, highlighting the complex applied anatomy associated with the surgical approaches to this area, specifically within the C5–C6 and C6–C7 intervertebral spaces. By providing detailed anatomical descriptions and considerations for emerging surgical interventions in equine surgery, such as the cervical foraminotomy, this study can enhance the surgical awareness of owners and equine veterinary surgeons and clinicians, facilitating better outcomes and postoperative recovery for the animals they treat. This informed approach not only aims to minimise surgical complications but also seeks to optimise the management of cervical spine pathologies in equines, ultimately contributing to improved equine health care practices (Figueiredo et al. 2006).

## 2 Material and Methods

### 2.1 Ethics Statement

This research study was conducted entirely under post‐mortem conditions and involved no procedures on live animals. Thus, it was not subject to the Animals (Scientific Procedures) Act 1986, Amendment Regulations (SI 2012/3039). Nonetheless, to ensure ethical oversight during this research. The study was reviewed and approved by the Royal Veterinary College's Clinical Research Ethical Review Board (CRERB) under URN 2025 2418‐A.

### 2.2 Specimen Collection

Four adult equine specimens were donated for this research. The specimens were fresh at the time of dissection, and both the head and neck were initially separated from the body at the C7–T1 junction to facilitate detailed anatomical dissection of the C5–C7 area, whilst ensuring the major structures were preserved. The dissections were performed consecutively on different days following the arrival of the specimens. The identification and descriptions of anatomical structures were performed in line with the anatomical description presented by Ashdown and Done (2011). Whenever necessary, a mechanical saw was utilised to excise the transverse processes of the cervical vertebrae, thereby improving visualisation and imaging of the deeper anatomical structures in the region.

### 2.3 Data Analysis

Concomitant with the dissections, images were obtained from the initial skin incisions through to the visualisation of the C5–C6 and C6–C7 IVF regions. Images were captured using the rear camera of an iPhone 13 smartphone (Apple Inc., Cupertino, CA, USA), featuring a 12‐MP sensor, and saved in JPEG format for labelling and editing. Schematic anatomical figures were created using the virtual software Ilustrae. Both Ilustrae and dissection figures were edited in Microsoft PowerPoint where applicable.

## 3 Results

The anatomical structures that the veterinary surgeon can expect to encounter from the skin through to the IVF in the region of C5–C7 are presented and described here in sequential order.

### 3.1 Skin and Muscles

**FIGURE 5:**
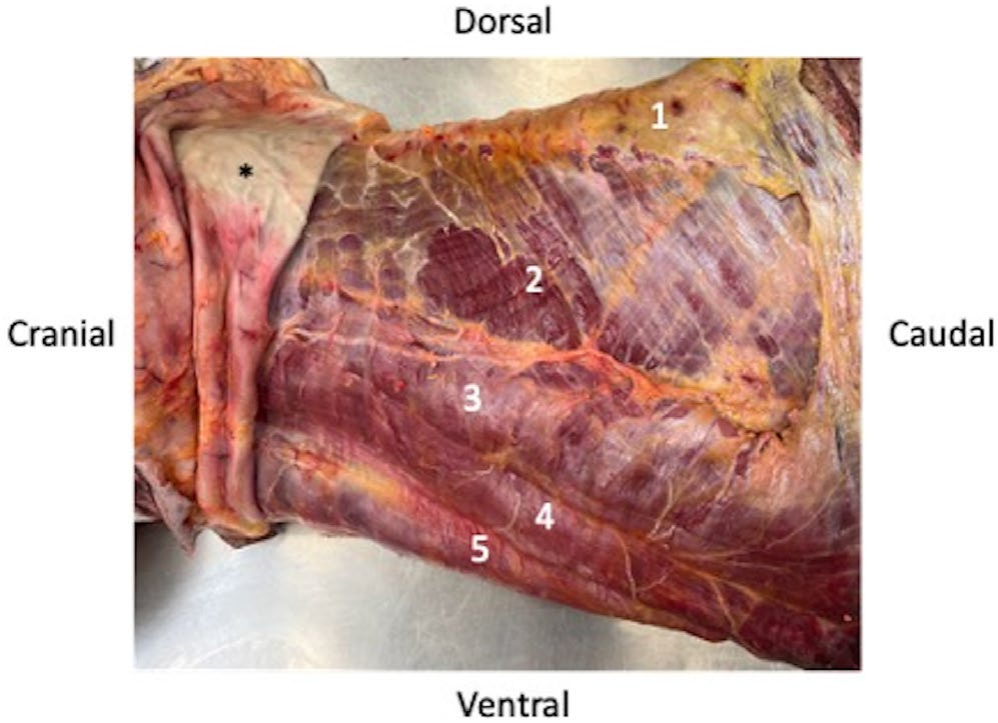
Ex vivo dissection of the equine neck specimen, left lateral view. Skin (*) reflected to the left (cranial) side, exposing subcutaneous and superficial fascia and superficial muscle layers. 1: Trapezius muscle, cervical part with fascia on top; 2: Splenius muscle; 3: Omotransversarius muscle; 4: Brachiocephalicus muscle; 5: Sternocephalicus muscle.

Initially, the equine neck specimens were dissected ex vivo and positioned in a left lateral view. The skin was carefully incised and reflected cranially to expose subcutaneous tissue and superficial fascia. Sequential blunt and sharp dissection separated the fascia to identify and preserve the trapezius, serratus ventralis cervicis, omotransversarius, cleidocephalicus, and sternocephalicus muscles (Figure 5).

**FIGURE 6:**
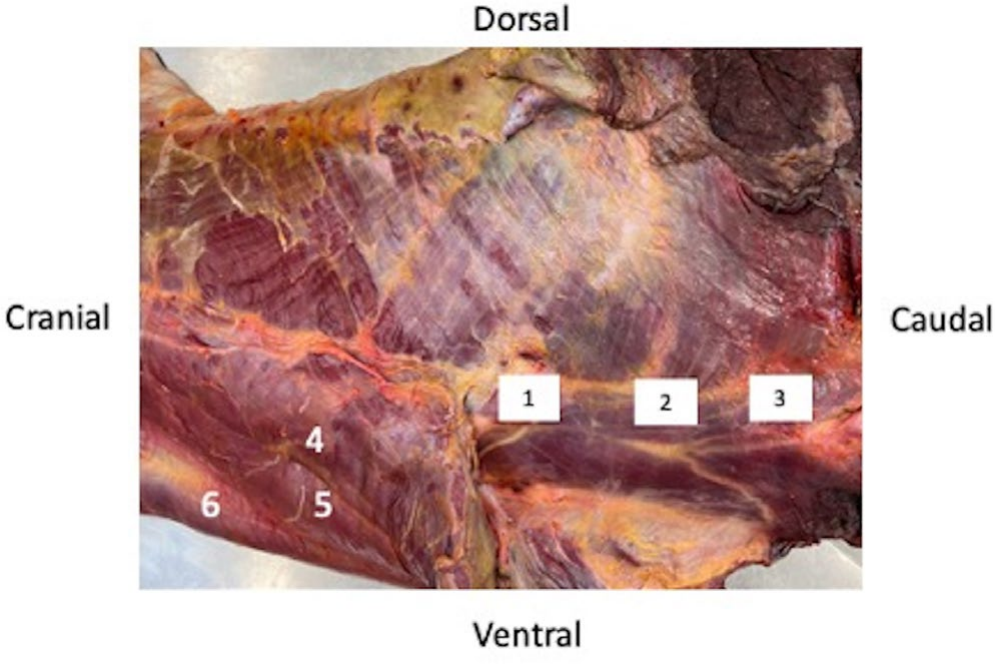
Ex vivo dissection of the equine neck specimen, left lateral view with reflection of omotransversarius muscle exposing the brachiocephalicus muscle. 1: Estimated C4–C5 intervertebral space; 2: Estimated C5–C6 intervertebral space; 3: Estimated C6–C7 intervertebral space; 4: Omotransversarius muscle; 5: Brachiocephalicus muscle; 6: Sternocephalicus muscle.

The omotransversarius muscle was carefully separated from surrounding fascia and reflected to fully expose the brachiocephalicus muscle. This further exposure allowed clearer visualisation of the cervical region. Anatomical landmarks were then estimated along the vertebral column, identifying the C4–C5, C5–C6, and C6–C7 intervertebral spaces for orientation (Figure 6).

**FIGURE 7:**
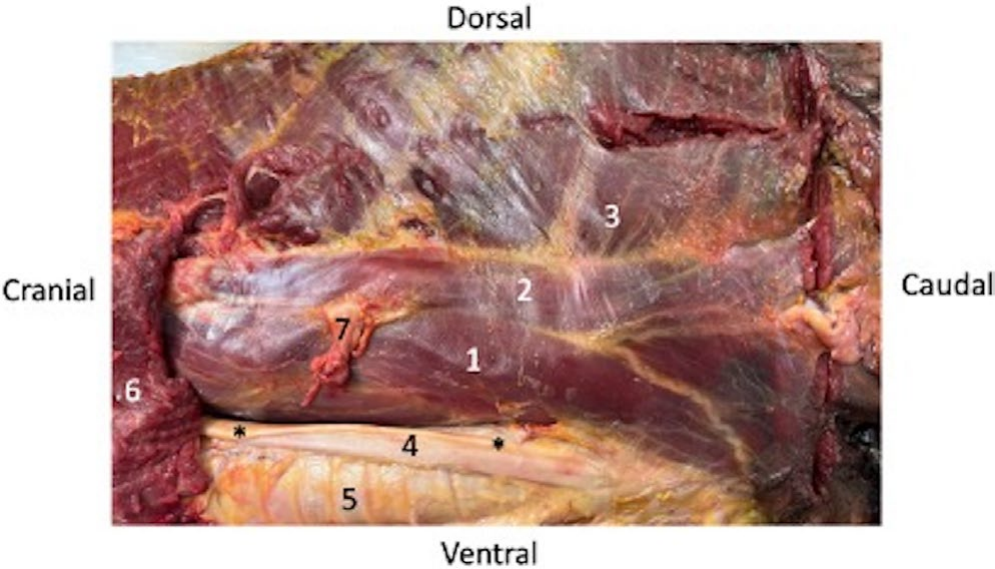
Ex vivo dissection of the equine neck specimen, left lateral view with reflection of the omotransversarius muscle exposing deeper muscles and structures. 1: Longus capitis muscle; 2: Scalenus ventralis muscle; 3: Serratus ventralis muscle; 4: Jugularis externa vein; 5: Trachea; 6: Brachiocephalicus muscle; 7: Cervicalis V nerve, ventralis branch; *, Oesophagus (collapsed).

The reflection of the omotransversarius and brachiocephalic muscles exposed deeper cervical structures, revealing underlying muscles, including the longus capitis, scalenus ventralis, and serratus ventralis. Important anatomical structures were also identified at this stage, such as the external jugular vein, trachea, and the ventral branch of the C5 cervical nerve, with the collapsed oesophagus visible dorsal to the trachea (Figure 7).

**FIGURE 8:**
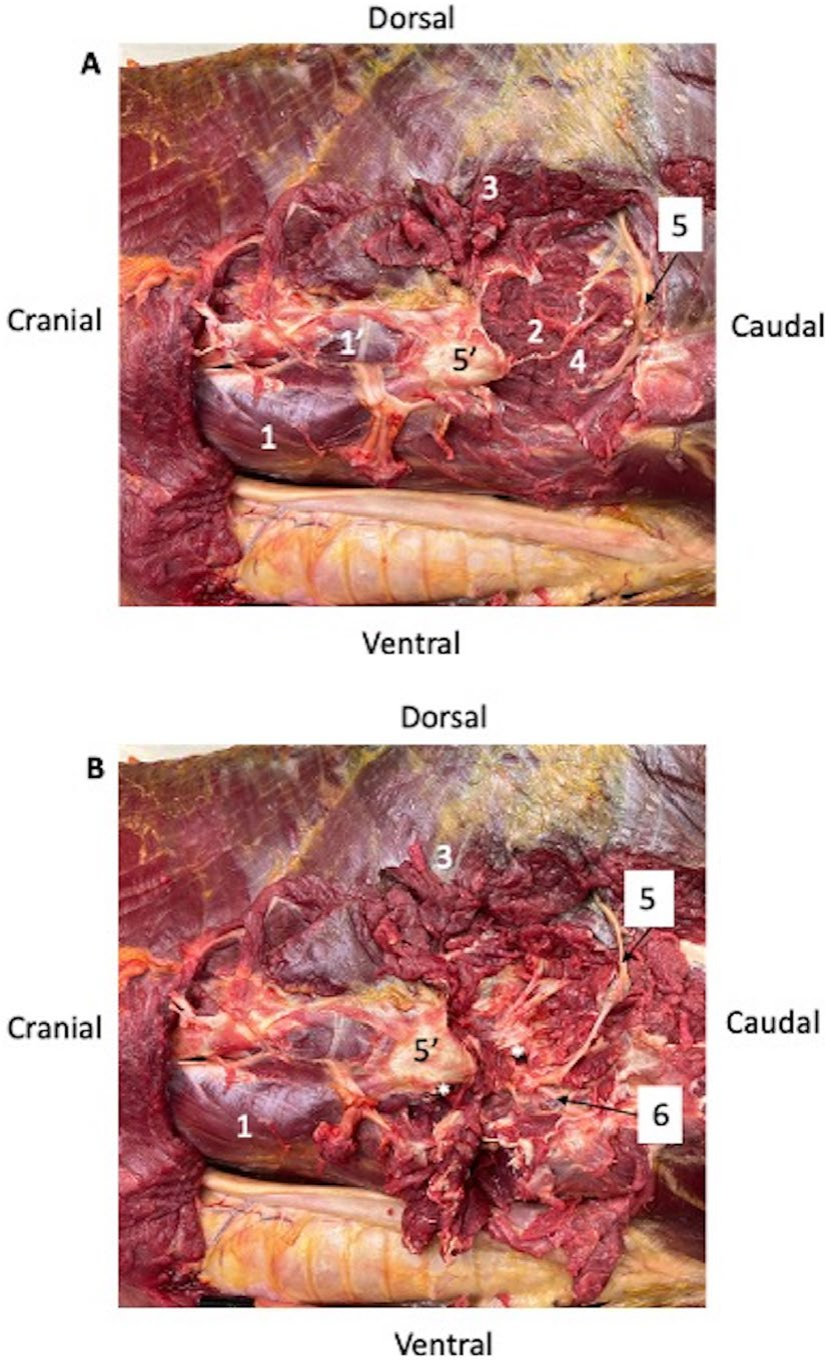
Ex vivo dissection of the equine neck with superficial muscles of the neck excised, exposing the deepest muscles, left lateral view. (A) Dissection of C6–C7 area with excision of scalenus medius muscle, exposing the longus colli muscle and intertransversarii ventralis cervicis muscle. (B) Dissection of C6–C7 area with excision of the longus colli muscle and intertransversarii ventralis cervicis muscle and exposure of intervertebral foramina (*). 1: Scalenus medius muscle (dorsal part); 1′: Scalenus medius muscle (ventral part); 2: Longus colli muscle; 3: Serratus ventralis cervicis muscle; 4: Intertransversarii ventralis muscle; 5′: Transverse process of C6; 5: Dorsal branches of the spinal nerves; 6: Ventral branch of C6 spinal nerve.

The dissection then progressed with complete excision of the omotransversarius, brachiocephalicus, and sternocephalicus muscles to expose the deepest muscle layers in the C6–C7 region. The scalenus medius muscle was carefully removed, revealing the longus colli and intertransversarii ventralis cervicis muscles adjacent to the transverse process of C6. Subsequent excision of these deeper muscles exposed the IVF and associated neural structures, including the dorsal branches of the spinal nerves and the ventral branch of the C6 spinal nerve (Figure 8).

### 3.2 The Intervertebral Foramina Structures

**FIGURE 9:**
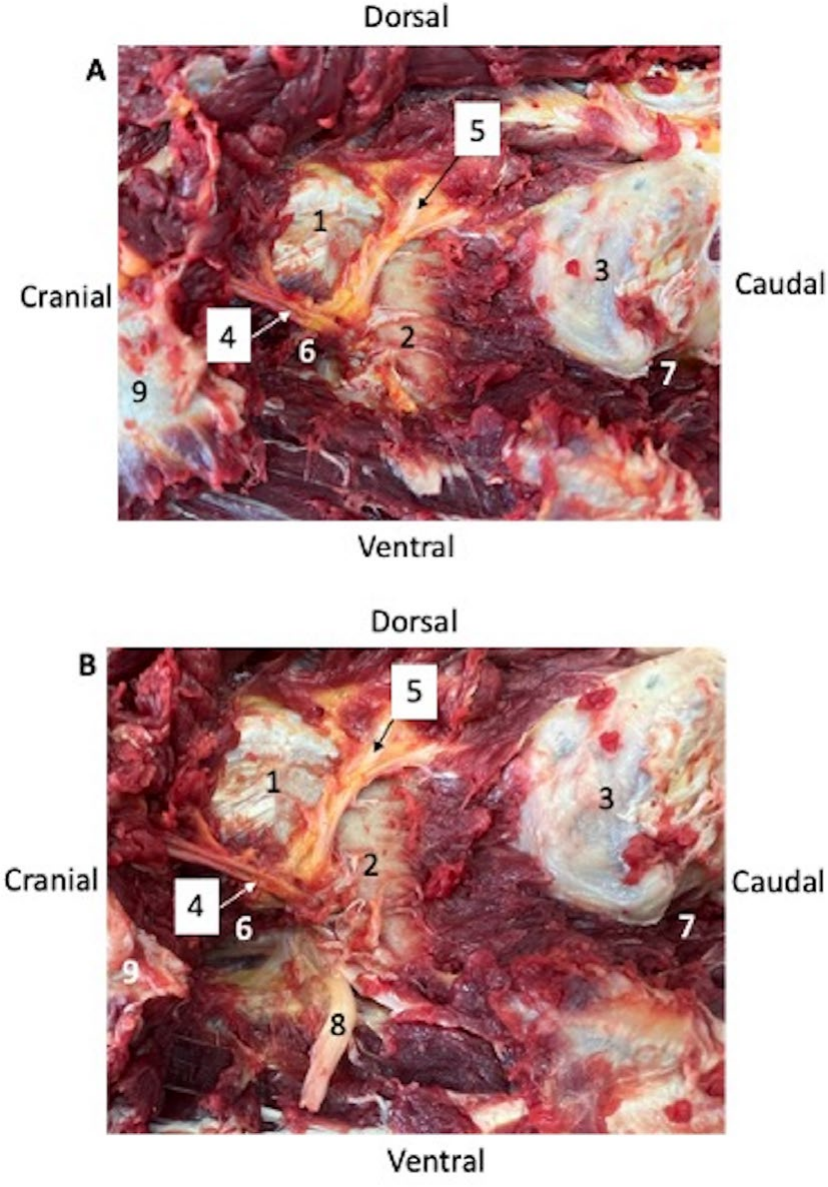
Ex vivo dissections of the equine neck at C5–C6 region. (A and B) 1: Caudal articular process of C5; 2: Ventral margin of the cranial articular process of C6; 3: Cranial articular process of C7; 4: Bundle containing the cutaneous branch of spinal nerve and ventral muscular branch of the vertebral artery; 5: Dorsal branch of cervicalis nerve VI; 6: Intervertebral foramen area of C5–C6; 7: Intervertebral foramen area of C6–C7; 8: Ventral branch of the spinal nerve; 9: Transverse process of C5.

Following the removal of the superficial and deep cervical muscles, the anatomical structures within the IVF became clearly visible. In the C5–C6 region, dissection exposed the articular processes of adjacent vertebrae and the transverse process of C5, allowing identification of the IVF areas. Neural and vascular structures were also visible at this stage, including the dorsal and ventral branches of the cervical spinal nerves and a bundle containing the cutaneous branch of the spinal nerve and a muscular branch of the vertebral artery (Figure 9).

**FIGURE 10:**
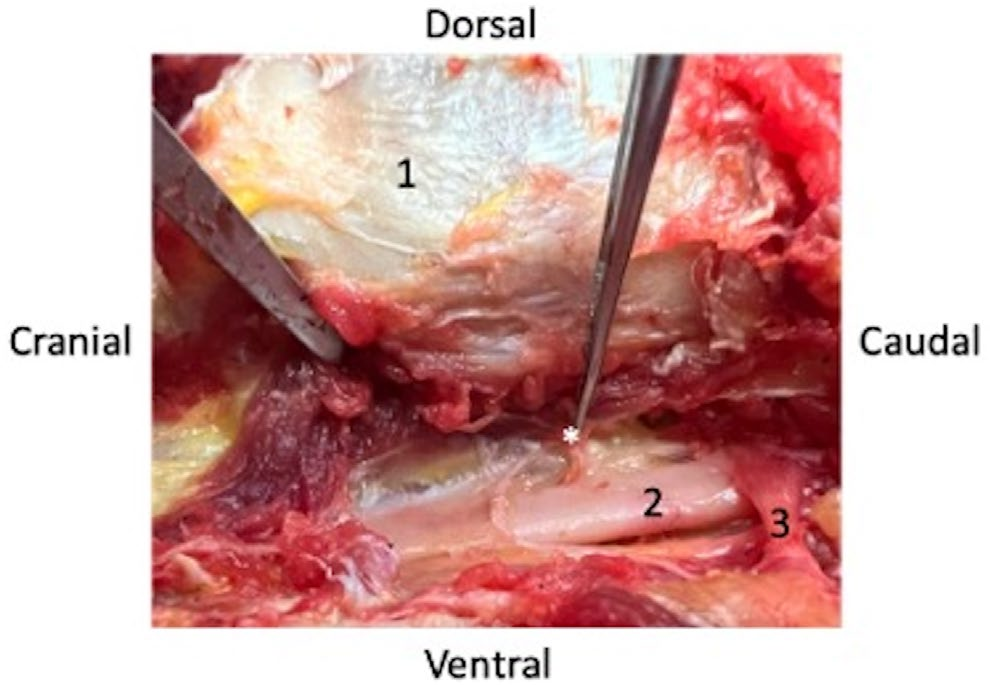
Left lateral view of the dissected transverse canal of the level of C5–C6, housing the vertebral artery, vein, and nerves. 1: Cranial articular process of C5; 2: Vertebral artery; 3: Spinal nerve; *, dorsal muscular branch of the vertebral artery.

Finally, the intervertebral structures at the C5–C6 level were clearly visualised, representing the key anatomical landmarks an equine surgeon should identify during a foraminotomy procedure. In the left lateral view, the dissected transverse canal revealed the vertebral neurovascular anatomical structures, including the vertebral artery and spinal nerve. The cranial articular process of C5 was also identified, along with the dorsal muscular branch of the vertebral artery (Figure 10).

## 4 Discussion

This descriptive study aimed to address a gap in the literature by providing a detailed anatomical characterisation of the equine C5–C6 and C6–C7 intervertebral spaces, with particular emphasis on the neurovascular structures within the IVF that are of clinical relevance to equine veterinary surgeons. Through systematic dissection and successive reflection of the superficial and deep cervical musculature, the regional anatomy of the IVF was exposed in a sequential manner, allowing clear visualisation of both vascular and neural structures and their spatial relationships. In this study, we drew particular attention to the vertebral arteries and vertebral veins within the intervertebral cervical region, as well as to the dorsal and ventral muscular branches of the vertebral arteries that arise in association with the IVF and supply the surrounding musculature of the neck.

This descriptive anatomical study has also aided in understanding the cervical musculature in relation to surgical procedures. A sequential dissection of the equine cervical musculature has been presented, highlighting the superficial and deep neck musculatures in the horse for a safe, effective foraminal intervention, such as decompression. Equine surgeons frequently navigate this region without a clear understanding of the muscular planes encountered along this anatomical access corridor to reach the IVF. According to the broader neurosurgical literature, precise anatomic dissection through the muscular layers is important to minimise collateral trauma, preserve postoperative function, and reduce complication risk in patients, principles that are well‐known in human neurosurgery, and yet directly applicable to equine cervical foraminotomy, for instance (Caballo et al. 2024; Kirnaz et al. 2021). Anatomical and clinical studies on cervical foraminotomy in humans and horses confirm the importance of the present study by showing that endoscopic or minimally invasive approaches rely on a deliberate pathway through multiple muscle groups to reach the intervertebral foramen while avoiding dorsal and ventral vascular and neural structures (Swagemakers et al. 2023; Vandenbulcke et al. 2024; Kirnaz et al. 2021). This pathfinding is dependent on knowing anatomical superficial and deep neck muscle planes and their relation to the uncovertebral joint, articular processes, and vertebral vessels.

This study also provides clinical insights and relevance in relation to equine cervical IVF anatomy. Understanding the detailed anatomy of the cervical IVF in horses, including the vertebral artery and vertebral vein as they traverse near the IVF, the exiting spinal nerves, and the surrounding venous plexuses, is critical for accurate clinical diagnosis and for improving surgical outcomes. The precise anatomical localisation of vascular and neural structures within, and adjacent to, the IVF informs both diagnostic imaging interpretation and the safety of invasive interventions in the area. For example, ultrasound‐guided approaches that can visualise the vertebral artery and vein in relation to the cervical spinal nerve roots between C5 and C7 enhance needle placement accuracy and minimise vascular injury during diagnostic or therapeutic injections (Johnson et al. 2021; Wood et al. 2021). The relevance of understanding and exploring the visualisation of the neurovascular topography of the equine neck through imaging techniques is further highlighted in equid cadaveric and ex vivo work that demonstrate reliable ultrasound visualisation and safe instrument passage ventral to vascular structures when the IVF is adequately identified (Johnson et al. 2021; Wood et al. 2021).

Clinically, foraminal narrowing or osteoarthritic remodelling of the caudal articular processes in the equine cervical vertebrae can compress exiting nerves, also known as radicular branches, causing forelimb lameness or neck pain that may be difficult to localise without imaging combined with precise anatomic knowledge of the IVF boundaries and the course of the vertebral vessels (Swagemakers et al. 2023; Fouquet et al. 2022; Wood et al. 2021). Endoscopic and minimally invasive surgeries, which are more commonly applied nowadays in equines for foraminal decompression, have demonstrated feasibility with careful attention to foraminal anatomy and neural‐vessel relationships, supporting improved outcomes when compression is confirmed and addressed with nerve root relief while avoiding vascular injury (Swagemakers et al. 2023).

A thorough understanding of the superficial and deep neck muscle groups in horses, together with the vasculature and nerve roots entering the intervertebral foramina between C5 and C7, is also essential for optimising both preoperative planning and postoperative recovery after foraminal decompression in horses. Muscular planes define safe surgical corridors to the intervertebral foramen, influence retraction and exposure, and dictate muscle‐sparing approaches that preserve neck stability and function (Swagemakers et al. 2023). The inadequate manipulation of the musculature and nerves across these anatomical planes can increase the risk of collateral muscle trauma, postoperative pain, and delayed return to performance, ultimately compromising welfare and athletic performance (Caballo et al. 2024).

The vascular and neural anatomical topography at any C5–C7 level can further inform intraoperative risk management and postoperative surveillance. The proximity of vertebral arteries, veins, and exiting spinal nerves within and around the IVF requires meticulous dissection and imaging‐guided navigation to minimise iatrogenic injury and ensure complete decompression while preserving neural function (Wood et al. 2021; Swagemakers et al. 2023). Thus, preoperative imaging that clearly shows muscle integrity, foraminal anatomy, and nearby vessels can enhance surgical planning, allow targeted, muscle‐sparing access, and support stable surgical reconstruction or fixation within the cervical region whenever indicated (Hou et al. 2022; Hojo et al. 2011). Postoperatively, the preservation of the musculature of the neck after an accurate foraminal decompression correlates with improved pain scores, faster return to work, and maintained cervical range of motion (Swagemakers et al. 2023). The integration of detailed muscular anatomy, regional vascular and neural relationships, and precise imaging‐guided techniques suggests the potential for improved prognostic outcomes and enhanced animal welfare in equine cervical procedures.

Although the variables of the present study were carefully assessed to achieve clear anatomical visualisation of the cervical and IVF structures in the horse, some limitations should be acknowledged. The number of the cadaveric specimens available for examination was limited, which may have constrained the observation of anatomical variation among individuals. Given the number of specimens, morphometric measurements of the neurovascular structures and surrounding anatomical landmarks were not appropriate. The structures within the intervertebral foramina were relatively small and delicate and located within narrow anatomical spaces, making accurate measurement during dissections technically challenging, yet of interest for future studies.

The detailed anatomical findings here suggest that precise visualisation of the intervertebral cervical vasculature and nerve roots within the IVF is indispensable for accurate diagnosis, risk‐minimised surgical interventions, and favourable surgical outcomes in equine patients. When anatomy is misunderstood, or imaging is suboptimal, there is an increased risk of vascular damage or incomplete decompression, potentially compromising prognosis and welfare. Future work should further quantify anatomic variations, including anatomical assessment of pathologic foramena, and standardise imaging protocols to optimise translational clinical practice in equine cervical spine surgery (Johnson et al. 2021; Swagemakers et al. 2023; Fouquet et al. 2022; Wood et al. 2021).

By enhancing their understanding of the relevant anatomy, surgeons can make informed decisions and avoid critical anatomical structures, thereby mitigating the risk of complications and enabling smoother surgical interventions. The primary objective of the study, to provide clear and comprehensive anatomical descriptions of the intervertebral foramina of the C5–C7 region in the horse, was achieved. The findings presented and detailed via sequential dissections serve as a vital resource for equine surgeons, guiding them through the intricacies of surgical procedures aimed at alleviating nerve compression syndromes. This anatomical description further emphasises the importance of precise anatomical knowledge in achieving rapid and uneventful recovery for equine patients following IVF surgery and sheds light on further studies that contribute positively to the welfare of horses suffering from spinal disorders, advancing the quality and efficacy of equine surgical care. The findings of this exploratory, descriptive study, combined with its clinical and surgical applications, offer valuable anatomical information that may inform future investigations, including imaging, morphometric, and surgical studies involving this clinically relevant and underexplored anatomical region.

## Conflicts of Interest

The authors declare no conflicts of interest.

## Data Availability

The data that support the findings of this study are available from the corresponding author upon reasonable request.

## References

[ahe70154-bib-0001] Ashdown, R. R. , and S. H. Done . 2011. Color Atlas of Veterinary Anatomy, Volume 2, The Horse. Elsevier Health Sciences.

[ahe70154-bib-0102] Bosch, K. , R. R. Zsoldos , A. Hartig , and T. Licka . 2025. “Motion Coupling at the Cervical Vertebral Joints in the Horse—An Ex Vivo Study Using Bone‐Anchored Markers.” Animals 15, no. 5: 2259. 10.3390/ani15152259. 40805049 PMC12345553

[ahe70154-bib-0002] Caballo, J. , A. Darden , S. Ahmad , and B. Boody . 2024. “The Cervical Intervertebral Foramen.” Clinical Spine Surgery a Spine Publication 38, no. 3: 97–102. 10.1097/bsd.0000000000001681. 39284205

[ahe70154-bib-0003] Clayton, H. M. , L. J. Kaiser , M. Lavagnino , and N. C. Stubbs . 2010. “Dynamic Mobilisations in Cervical Flexion: Effects on Intervertebral Angulations.” Equine Veterinary Journal 42, no. s38: 688–694. 10.1111/j.2042-3306.2010.00196.x. 21059082

[ahe70154-bib-0004] Corraretti, G. , J. Vandeweerd , F. Hontoir , K. Vanderperren , and K. Palmers . 2020. “Anatomy and Ultrasound‐Guided Injection of the Medial Branch of the Dorsal Ramus of the Cervical Spinal Nerves in the Horse: A Cadaveric Study.” Veterinary and Comparative Orthopaedics and Traumatology 33, no. 6: 377–386. 10.1055/s-0040-1714301. 32777844

[ahe70154-bib-0005] Dias, F. G. G. , V. T. D. S. Almeida , V. M. R. Ramos , et al. 2024. “Comparison Between Cervical Vertebrae of Man and of the Domestic Animals.” Cuadernos de Educación y Desarrollo 16, no. 2: e3159. 10.55905/cuadv16n2-020.

[ahe70154-bib-0006] Elliott, R. , and C. A. Boucher . 2018. “Radicular Compression of the Nerve Root in the Intervertebral Foramen by an Intervertebral Cervical Disc Extrusion of a Dachshund.” Veterinary Record Case Reports 6, no. 2: e000646. 10.1136/vetreccr-2018-000646.

[ahe70154-bib-0007] Figueiredo, E. G. , M. C. D. L. Cruz , N. Theodore , P. Deshmukh , and M. C. Preul . 2006. “Modified Cervical Laminoforaminotomy Based on Anatomic Landmarks Reduces Need for Bony Removal.” Min—Minimally Invasive Neurosurgery 49, no. 1: 37–42. 10.1055/s-2006-932146. 16547881

[ahe70154-bib-0008] Fouquet, G. , G. Abbas , J. P. Johnson , et al. 2022. “Ultrasound‐Guided Injection Technique of the Equine Cervical Nerve Roots.” Frontiers in Veterinary Science 9: 992208. 10.3389/fvets.2022.992208. 36387391 PMC9644134

[ahe70154-bib-0009] Gilchrist, R. V. 2002. “Anatomy of the Intervertebral Foramen.” Pain Physician 5, no. 4: 372–378. 10.36076/ppj.2002/5/372. 16886015

[ahe70154-bib-0011] Haussler, K. , R. Pool , and H. Clayton . 2019. “Characterization of Bony Changes Localized to the Cervical Articular Processes in a Mixed Population of Horses.” PLoS One 14, no. 9: e0222989. 10.1371/journal.pone.0222989. 31557207 PMC6762202

[ahe70154-bib-0012] Hojo, Y. , M. Ito , K. Abumi , et al. 2011. “A Late Neurological Complication Following Posterior Correction Surgery of Severe Cervical Kyphosis.” European Spine Journal 20, no. 6: 890–898. 10.1007/s00586-010-1590-8. 20936306 PMC3099150

[ahe70154-bib-0013] Hou, G. L. , C. M. Chen , K. T. Chen , et al. 2022. “Circumferential Decompression Technique of Posterior Endoscopic Cervical Foraminotomy.” BioMed Research International 2022, no. 1: 5873333. 10.1155/2022/5873333. 35111847 PMC8803431

[ahe70154-bib-0014] Johnson, J. , T. Vinardell , and F. David . 2021. “Ultrasound‐Guided Injections of the Equine Head and Neck: Review and Expert Opinion.” Journal of Equine Science 32, no. 4: 103–115. 10.1294/jes.32.103. 35023988 PMC8731684

[ahe70154-bib-0015] Kirnaz, S. , H. Gebhard , T. Wong , et al. 2021. “Intraoperative Image Guidance for Cervical Spine Surgery.” Annals of Translational Medicine 9, no. 1: 93. 10.21037/atm-20-1101. 33553386 PMC7859826

[ahe70154-bib-0016] Kraan, G. , P. V. Hoogland , and P. Wuisman . 2009. “Extraforaminal Ligament Attachments of the Thoracic Spinal Nerves in Humans.” European Spine Journal 18, no. 4: 490–498. 10.1007/s00586-009-0881-4. 19165508 PMC2899458

[ahe70154-bib-0017] Long, M. , C. Dürnberger , F. Jenner , Z. Kelemen , U. Auer , and H. Grimm . 2022. “Quality of Life Within Horse Welfare Assessment Tools: Informing Decisions for Chronically Ill and Geriatric Horses.” Animals 12, no. 14: 1822. 10.3390/ani12141822. 35883370 PMC9311870

[ahe70154-bib-0018] McElroy, A. , A. M. Rashmir‐Raven , J. M. Manfredi , et al. 2019. “Evaluation of the Structure of Myodural Bridges in an Equine Model of Ehlers‐Danlos Syndromes.” Scientific Reports 9, no. 1: 9978. 10.1038/s41598-019-46444-w. 31292490 PMC6620297

[ahe70154-bib-0019] Pasquini, C. 1991. Atlas of Equine Anatomy: Regional Approach. 3rd ed. Sudz Publishing.

[ahe70154-bib-0020] Pepe, M. , M. Angelone , R. Gialletti , S. Nannarone , and F. Beccati . 2014. “Arthroscopic Anatomy of the Equine Cervical Articular Process Joints.” Equine Veterinary Journal 46, no. 3: 345–351. 10.1111/evj.12112. 23742017

[ahe70154-bib-0021] Prange, T. , B. Shrauner , and A. Blikslager . 2015. “Epiduroscopy of the Lumbosacral Vertebral Canal in the Horse: Technique and Endoscopic Anatomy.” Equine Veterinary Journal 48, no. 1: 125–129. 10.1111/evj.12470. 26084359

[ahe70154-bib-0221] Rombach, N. , N. C. Stubbs , and H. M. Clayton . 2014. “Prevalence of Osseous Pathology in the Articular Process Articulations in the Equine Cervical and Cranial Thoracic Vertebrae.” Equine Veterinary Journal 46: 10–10. 10.1111/evj.12470.24329582

[ahe70154-bib-0022] Schmaltz, R. 1928. Anatomie des Pferdes: In den Grenzen der Vorlesung Dargestellt. 2nd ed. Schoetz.

[ahe70154-bib-0023] Singh, B. 2018. Dyce, Sack, and Wensing's Textbook of Veterinary Anatomy. Saunders.

[ahe70154-bib-0024] Sioutas, G. S. , and S. Kapetanakis . 2016. “Clinical Anatomy and Clinical Significance of the Cervical Intervertebral Foramen: A Review.” Folia Morphologica 75, no. 2: 143–148. 10.5603/fm.a2015.0096. 26542962

[ahe70154-bib-0025] Swagemakers, J. , P. Daele , and M. Mageed . 2023. “Percutaneous Full Endoscopic Foraminotomy for Treatment of Cervical Spinal Nerve Compression in Horses Using a Uniportal Approach: Feasibility Study.” Equine Veterinary Journal 55, no. 5: 788–797. 10.1111/evj.13919. 36572912

[ahe70154-bib-0026] Vandenbulcke, A. , A. Sanjurjo , A. Rougemont , S. Boudabbous , and R. Maduri . 2024. “Subaxial Cervical Foraminal Chondromas: Case‐Based Discussion on Surgical Management.” Neurosurgical Review 47, no. 1: 834. 10.1007/s10143-024-03065-w. 39489866 PMC11532321

[ahe70154-bib-0027] Wood, A. , M. Sinovich , J. Prutton , and R. Parker . 2021. “Ultrasonographic Guidance for Perineural Injections of the Cervical Spinal Nerves in Horses.” Veterinary Surgery 50, no. 4: 816–822. 10.1111/vsu.13610. 33751588

